# Invitation for an International Medical and Scientific Exhibition

**Published:** 1890-04

**Authors:** 


### INVITATION FOR AN INTERNATIONAL MEDICAL AND SCIENTIFIC EXHIBITION.

In connection with the Tenth International Medical Congress to be held in Berlin, between the Fourth and Tenth of August, there is to be an International Medical and Scientific Exhibition. The exhibits will be of an exclusively scientific nature as follows:

New or improved scientific instruments and apparatus for biological and strictly medical purposes, inclusive of apparatus for photography and spectral analysis as far as applicable to medicine.

New objects and preparations in pharmacological chemistry and pharmacy.

New foods.

New or improved instruments subservient to any of the departments of medicine, including electrotherapy.

New plans and models for hospitals, convalescent homes, and disinfecting and bathing institutions and apparatus.

New arrangements for nursing, including transportation, baths, etc.

New apparatus in hygiene.

Applications or inquiries inscribed "Ausstellungs-Angelegenheit," and accompanied with a printed card containing the name and address of a firm thus applying, ought to be directed to the Secretary General, Dr. O. Lassar, Carltrasse, No. 19, Berlin, N. W., Germany.

R. VIRCHOW, President.

E. VON BERGMANN, E. LEYDEN, W. WALDEYER, Vice-Presidents.

O. LASSAR, Secretary General.

